# Impact of Thoracic Duct Resection on Postoperative Body Composition Trajectory After Oesophagectomy: A Prospective Cohort Study

**DOI:** 10.1002/jcsm.70209

**Published:** 2026-02-10

**Authors:** Tae Hee Hong, Young Ho Yang, Ha Eun Kim, Byung Jo Park, Chang Young Lee, Jin Gu Lee, Dae Joon Kim

**Affiliations:** ^1^ Department of Thoracic and Cardiovascular Surgery Yonsei University College of Medicine, Severance Hospital Seoul Republic of Korea

**Keywords:** bioelectrical impedance analysis, body composition, oesophagectomy, sarcopenia, skeletal muscle mass, thoracic duct resection

## Abstract

**Background:**

Thoracic duct resection (TDR) is frequently performed during radical oesophagectomy to improve locoregional control in oesophageal squamous cell carcinoma (ESCC). However, its impact on postoperative body composition—particularly skeletal muscle mass—remains unclear. This study aimed to evaluate the extent and temporal pattern of postoperative changes in adiposity‐ and sarcopenia‐related indices following TDR.

**Methods:**

In this prospective cohort study, 347 patients with ESCC who underwent curative oesophagectomy between May 2018 and June 2022 were included. Patients were classified into a TDR group (*n* = 288) and a thoracic duct preservation group (*n* = 59). Body composition was assessed using bioelectrical impedance analysis (BIA) at six time points: preoperatively and 1, 2, 3, 6 and 12 months postoperatively, yielding 1925 measurements. Metrics analysed included body mass index (BMI), fat mass index (FMI), skeletal muscle mass index (SMI) and fat‐free mass index (FFMI). Sensitivity analysis was performed using 1:1 propensity score matching (PSM), based on key clinical variables.

**Results:**

Median age was 64 years, and ~90% of patients were male in both groups. Baseline operative variables were comparable between groups, including operative time (485 vs. 478 min), total lymph nodes (66 vs. 63) and complication rates (30% vs. 32%). BMI and FMI declined gradually over 12 months with no significant between‐group differences (BMI at 12 months: TDR vs. preservation, 21.0 vs. 20.6 kg/m^2^; *p* = 0.809). In contrast, SMI and FFMI showed significant early postoperative declines, with more pronounced reductions in the TDR group during the first 3 months (SMI: −11.2% vs. −8.1%, *p* = 0.036). These early differences attenuated after PSM but remained directionally consistent. Recovery of muscle mass began around postoperative month 3, and by 12 months, sarcopenia‐related indices were comparable between groups (SMI: *p* = 0.343; FFMI: *p* = 0.733). Subgroup analysis in patients with clinical stage I disease revealed similar patterns, suggesting that the observed muscle loss may reflect procedure‐related effects, independent of tumour burden. Exploratory nutritional markers—including albumin, lymphocyte count and cholesterol—showed no significant intergroup differences at any interval.

**Conclusions:**

This is the largest study to date to assess longitudinal body composition changes after TDR using serial BIA. TDR was associated with a greater decline in sarcopenia‐related indices, particularly within the first 3 months. These effects were transient, reversible and reproducible in early‐stage patients. Our findings support the oncologic role of TDR while underscoring the importance of early nutritional and rehabilitative care.

## 1 Introduction

Radical oesophagectomy remains a cornerstone treatment for resectable oesophageal cancer, particularly oesophageal squamous cell carcinoma (ESCC) [1, 2]. The procedure aims to achieve curative resection by removing the primary tumour along with regional lymphatic structures, often including thoracic duct resection (TDR) to ensure comprehensive nodal clearance and optimal locoregional control [3, 4]. Although TDR offers potential oncologic benefits, its physiological impact on postoperative recovery—especially in terms of nutritional status and body composition—remains inadequately characterized [5].

The thoracic duct is the largest lymphatic vessel in the body and plays a central role in lipid absorption, protein transport and fluid homeostasis [6]. Its resection may disrupt these essential functions, potentially leading to measurable alterations in body composition, such as reductions in skeletal muscle and fat mass. These changes are clinically relevant, as postoperative body composition metrics are closely linked to recovery speed, functional capacity and long‐term prognosis [5, 7]. Although previous studies have addressed perioperative complications related to TDR, data on its impact on nutritional and metabolic recovery are limited and primarily derived from small‐scale retrospective analyses [7, 8, 9].

To address this gap, the present study aimed to evaluate the longitudinal trajectory of body composition following TDR in over 300 patients undergoing radical oesophagectomy for ESCC. Specifically, we sought to quantify the extent and duration of postoperative changes in muscle and fat compartments and to assess whether these effects are transient or sustained. By doing so, we aimed to refine the clinical indications for TDR and inform perioperative management strategies. Our findings may support the integration of early nutritional intervention and rehabilitation planning, particularly in patients with early‐stage disease, where balancing oncologic benefit and functional preservation is crucial.

## 2 Methods

### 2.1 Study Design and Patients

**FIGURE 1:**
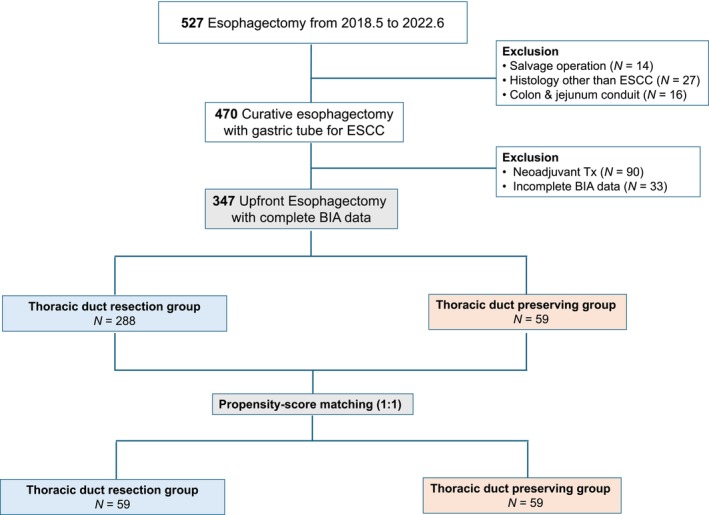
Flowchart of patients included in the study.

This longitudinal cohort study was conducted at Severance Hospital, Yonsei University College of Medicine. From May 2018 to June 2022, a total of 470 patients who underwent curative oesophagectomy with gastric tube reconstruction for oesophageal cancer were initially identified. Exclusion criteria included neoadjuvant treatment (*n* = 90), incomplete body composition data (*n* = 33), salvage operations (*n* = 14), histological findings other than ESCC (*n* = 27) and reconstruction using colon or jejunum conduits (*n* = 16). After applying these exclusion criteria, 347 patients were included in the final analysis, with 288 undergoing TDR and 59 undergoing thoracic duct preservation (Figure 1). Cancer staging was determined according to the American Joint Committee on Cancer (AJCC) 8th edition. This study was conducted in accordance with the Declaration of Helsinki and was approved by the institutional review board (IRB: 4‐2020‐1335).

### 2.2 Surgical Procedure

As previously described [10], skeletonizing en bloc oesophagectomy (SEBE) has been our standard procedure for resectable ESCC since 2017, aiming for more radical dissection of tumours and regional lymph nodes. SEBE included en bloc removal of the oesophagus and locoregional lymph nodes from the hiatus to the apex, along with bilateral pleura and soft tissues covering the descending thoracic aorta, the trachea and main bronchi and the pericardium. Lymph nodes along the left RLN and aortopulmonary window were dissected separately to protect the nerve. In the TDR group, the TD was resected en bloc with the specimen throughout its course and clipped at the level of the T10–11 vertebra. In the preservation group, the TD was not dissected, whereas other components of oesophagectomy remained the same as the TDR group.

The decision to perform TDR was at the discretion of the operator. At our institution, TDR was generally considered for ESCC patients with clinical stage T2 or higher (tumour invading the proper muscle) or clinical node‐positive (N+) disease. In contrast, for clinical stage 1 cases, TDR was selectively performed based on specific clinical considerations, including tumour extent, intraoperative frozen section examinations for metastatic lymph nodes, underlying comorbidities and the surgeon's assessment of potential postoperative risks.

### 2.3 Perioperative Nutritional and Rehabilitation Protocol

All patients received standardized perioperative nutritional and rehabilitation care that was uniformly applied to both the TDR and preservation groups. Early enteral nutrition was initiated on postoperative days (POD) 2–3 via a jejunostomy tube, with caloric and protein targets of 25–30 kcal/kg/day and ≥ 1.2 g/kg/day. Oral intake was resumed after POD7, once the absence of anastomotic leakage was confirmed, and then advanced according to a structured stepwise diet progression. Rehabilitation therapy followed a unified institutional protocol, beginning on POD1 with early mobilization, incentive spirometry and progressive ambulation, which was further intensified before discharge and continued with home‐based walking and light resistance exercises after discharge. Access to nutritional support and rehabilitation guidance remained consistent across both groups throughout the observation period.

### 2.4 Data Collection and Body Composition Metrics

Body composition was assessed using a bioelectrical impedance analysis (BIA) device (InBody scale; InBody Co. Ltd., Seoul, Korea). Measurements were obtained preoperatively and at 1, 2, 3, 6 and 12 months postoperatively. The following indices were analysed:

- Body mass index (BMI, kg/m^2^): Total body weight divided by height squared; a general measure reflecting both fat and lean mass.
- Fat mass index (FMI, kg/m^2^): Fat mass divided by height squared; a fat‐specific indicator of adiposity.
- Fat‐free mass index (FFMI, kg/m^2^): (Total body weight − fat mass) divided by height squared; a muscle‐related indicator reflecting non‐fat body components.
- Skeletal muscle mass index (SMI, kg/m^2^): Skeletal muscle mass divided by height squared; a specific marker of muscularity.

For analysis, BMI and FMI were classified as adiposity‐related metrics, whereas FFMI and SMI were considered sarcopenia‐related (muscle‐related) indices, allowing for focused evaluation of fat versus muscle changes over time.

### 2.5 Outcome Measures and Statistical Analysis

The primary outcome was the change in body composition metrics over the postoperative period. Secondary outcomes included differences in recovery times and patterns between the TDR and preservation groups. Temporal trends in SMI and FFMI were analysed to identify group‐specific patterns. For exploratory purposes, nutritional laboratory markers—including albumin, total lymphocyte count, total cholesterol, prognostic nutrition index (PNI) and Controlling Nutritional Status (CONUT) score—were also evaluated. Because laboratory testing was not part of the original study protocol, these data were available only in a subset of patients (~60%–70%). Analyses were performed in a pair‐wise manner, including only patients with results available at both time points for each interval (pre vs. 1–3 months and 1–3 vs. 6–12 months comparisons).

To reduce potential selection bias arising from surgeon‐dependent choice of TDR, we additionally performed propensity score matching (PSM). Matching was conducted using 1:1 nearest‐neighbour matching without replacement (calliper = 0.2 SD of the logit of the propensity score), based on clinically relevant covariates: age, sex, clinical stage, tumour location, type of oesophagectomy and surgical approach.

Baseline characteristics and operative outcomes were compared using chi‐square tests for categorical variables and independent *t*‐tests for continuous variables. Longitudinal changes in body composition were analysed using repeated measures ANOVA. A *p*‐value of < 0.05 was considered statistically significant. All statistical analyses were performed using the R statistical programming language (Version 4.0.3, R Foundation for Statistical Computing, Vienna, Austria).

## 3 Results

### 3.1 Baseline Characteristics

The TDR and thoracic duct preservation groups exhibited comparable baseline characteristics in terms of age, sex distribution and tumour location (Table 1). The median age was 64 years in both groups (*p* = 0.533), and the proportion of male patients was similarly high (92% in the preservation group vs. 90% in the TDR group, *p* = 0.707).

**TABLE 1:** Baseline characteristics of thoracic duct resection (TDR) group and the preservation group.

Variables	Before matching			After matching			
	Preservation group^a^	TDR group^a^	*p*	Preservation group^a^	TDR group^a^	*p*	SMD
	(*n* = 59)	(*n* = 288)		(*n* = 59)	(*n* = 59)		
Age, year	64 [59, 71]	64 [58, 69]	0.533	64 [59, 71]	64 [59, 69]	0.532	0.123
Sex			0.707			0.436	0.072
Female	5 (8.5%)	29 (10%)		5 (8.5%)	2 (3.4%)		
Male	54 (92%)	259 (90%)		54 (92%)	57 (96.6%)		
Tumour location			0.331			0.990	0.042
Cervical	2 (3.4%)	2 (0.7%)		2 (3.4%)	2 (3.4%)		
Upper thoracic	8 (14%)	40 (14%)		8 (14%)	8 (14%)		
Middle thoracic	25 (42%)	135 (47%)		25 (42%)	27 (46%)		
Lower thoracic	21 (36%)	103 (36%)		21 (36%)	20 (34%)		
Oesophago‐gastric junction	3 (5.1%)	8 (2.8%)		3 (5.1%)	2 (3.4%)		
Preoperative tumour grade			0.957			0.869	0.072
G1	3 (5.1%)	21 (7.3%)		3 (5.1%)	4 (6.8%)		
G2	29 (49%)	139 (48%)		29 (49%)	27 (45.8%)		
G3	8 (14%)	43 (15%)		8 (14%)	6 (10.2%)		
GX	19 (32%)	85 (30%)		19 (32%)	22 (37.3%)		
Clinical T stage, 8th AJCC			< 0.001			0.280	0.094
T1a	20 (34%)	42 (15%)		20 (34%)	11 (18.6%)		
T1b	29 (49%)	116 (40%)		29 (49%)	33 (55.9%)		
T2	3 (5.1%)	60 (21%)		3 (5.1%)	7 (11.9%)		
T3	7 (12%)	70 (24%)		7 (12%)	8 (13.6%)		
Clinical N stage, 8th AJCC			0.013			0.552	0.063
NO	51 (86%)	195 (68%)		51 (86%)	53 (89.8%)		
N1	7 (12%)	77 (27%)		7 (12%)	4 (6.8%)		
N2	1 (1.7%)	16 (5.6%)		1 (1.7%)	2 (3.4%)		
Clinical stage, 8th AJCC			< 0.001			0.953	0.034
IA	20 (34%)	61 (21%)		20 (34%)	20 (33.9%)		
IB	27 (46%)	71 (25%)		27 (46%)	27 (45.8%)		
IIA	2 (3.4%)	35 (12%)		2 (3.4%)	2 (3.4%)		
IIB	5 (8.5%)	80 (28%)		5 (8.5%)	6 (10.2%)		
IIIA	3 (5.1%)	30 (10%)		3 (5.1%)	3 (5.1%)		
I11B	2 (3.4%)	11 (3.8%)		2 (3.4%)	1 (1.7%)		
Type of oesophagectomy			0.064			0.604	0.024
Ivor–Lewis	2 (3.4%)	3 (1.0%)		2 (3.4%)	2 (3.4%)		
McKeown	56 (95%)	285 (99%)		56 (95%)	57 (96.6%)		
Transhiatal	1 (1.7%)	0 (0%)		1 (1.7%)	0 (0.0%)		
Surgical approach			0.078			0.642	0.054
Open	8 (14%)	44 (15%)		8 (14%)	5 (8.5%)		
Laparoscopic	5 (8.5%)	47 (16%)		5 (8.5%)	47 (79.7%)		
Robotic	46 (78%)	197 (68%)		46 (78%)	7 (11.9%)		

Abbreviations: AJCC, American Joint Committee on Cancer; TDR, thoracic duct resection.

^a^ Median (IQR); *n* (%).

Tumour location and histologic grade were also similar, with no statistically significant differences (*p* = 0.331 and *p* = 0.957, respectively). However, clinical T stage (according to 8th AJCC system) differed significantly between the groups (*p* < 0.001): The preservation group had a higher proportion of early‐stage tumours (T1: 83% vs. 55%), whereas the TDR group included more patients with advanced tumours (T2–T4: 45% vs. 17%). Lymph node involvement (clinical N stage) was also significantly different (*p* = 0.013), with more node‐negative cases in the preservation group (86% vs. 68%). Clinical stage group showed a similar pattern, with early‐stage disease (IA–IB) more prevalent in the preservation group (80% vs. 46%, *p* < 0.001) and more advanced‐stage disease (IIA–IIIB) in the TDR group.

Regarding surgical technique, the McKeown procedure was the most commonly performed in both groups (95% vs. 99%, *p* = 0.064). The operative approach for the thoracic phase showed no significant difference (*p* = 0.078), with robotic‐assisted surgery being the most frequently used method (78% in the preservation group vs. 68% in the TDR group). These findings reflect comparable baseline demographic and histopathologic characteristics while also highlighting institutional practices of selecting TDR based on clinical staging.

After PSM, these baseline imbalances were effectively minimized. Key demographic, clinical and surgical variables were well balanced between groups (most SMD < 0.1), and none of the previously noted differences remained statistically significant. This ensures that subsequent comparisons in postoperative trajectories were evaluated in cohorts with comparable baseline characteristics.

### 3.2 Operative Outcomes

The median operative time and estimated blood loss were comparable between the TDR and preservation groups (operative time: 485 vs. 478 min, *p* = 0.378; blood loss: 100 mL in both groups, *p* = 0.474; Table 2). The number of harvested lymph nodes did not differ significantly between groups across cervical, thoracic, abdominal or total regions (total lymph nodes: 66 vs. 63, *p* = 0.183). Rates of R0 resection were similarly high in both groups (99% vs. 98%, *p* = 0.429). Postoperative complications of Grades 3 and 4 occurred in 30% of the TDR group and 32% of the preservation group (*p* = 0.686), with pneumonia being the most frequent complication. The incidence of chylothorax was slightly higher in the preservation group (6.8% vs. 4.5%, *p* = 0.687), but the difference was not statistically significant. Median length of hospital stay (16 vs. 17 days, *p* = 0.728) and ICU stay (2 days in both groups, *p* = 0.590) were similar between the two groups.

**TABLE 2:** Comparison of operative outcomes between the thoracic duct resection (TDR) group and the preservation group.

Variables	Before matching			After matching			
	Preservation group^a^	TDR group^a^	*p*	Preservation group^a^	TDR group^a^	*p*	SMD
	(*n* = 59)	(*n* = 288)		(*n* = 59)	(*n* = 59)		
Resection margin			0.429			1.00	0.000
RO	58 (98%)	286 (99%)		58 (98%)	58 (98%)		
R1	1 (1.7%)	2 (0.7%)		1 (1.7%)	1 (1.7%)		
Harvested lymph nodes	63 (47, 79)	66 (53, 79)	0.183	63 (47, 79)	63 (49, 77)	0.554	0.154
Cervical	18 (13, 27)	19 (15, 25)		18 (13, 27)	18 (13, 24)		
Thoracic	25 (20, 27)	27 (22, 30)		25 (20, 27)	27 (23, 29)		
Abdominal	19 (14, 25)	20 (16, 24)		19 (14, 25)	20 (16, 23)		
Operative time (min)	478 (436, 554)	485 (445, 526)	0.378	478 (436, 554)	476 (441, 517)	0.445	−0.067
Estimated blood loss (ml)	100 (50, 300)	100 (50, 200)	0.474	100 (50, 300)	90 (20, 210)	0.922	0.080
Postsurgical complications (%)							
Any complications, Gr 3–4	19 (32%)	89 (30%)	0.686	19 (32%)	19 (32%)	1.00	0.000
Bleeding	0 (0.0%)	2 (0.7%)	0.363	0 (0.0%)	1 (1.7%)	0.320	−0.186
Chylothorax	4 (6.8%)	13 (4.5%)	0.687	4 (6.8%)	5 (8.5%)	0.780	−0.064
Pneumonia	5 (8.5%)	24 (8.3%)	0.99	5 (8.5%)	6 (10.2%)	0.820	−0.058
Anastomosis site leakage	4 (6.8%)	28 (9.7%)	0.642	4 (6.8%)	5 (8.5%)	0.800	−0.064
Length of hospital stay (days)	17 (12, 25)	16 (12, 23)	0.728	17 (12, 25)	18 (14, 23)	0.155	−0.223
Length of ICU stat (dats)	2 (1, 4)	2 (1, 3)	0.59	2 (1, 4)	2.5 (1.2, 3.7)	0.241	−0.157

Abbreviations: ICU, intensive care unit; TOR, thoracic duct resection.

^a^ Median (IQR); *n* (%).

After matching, operative outcomes remained comparable. Operative time, blood loss, total lymph nodes harvested, major complications, R0 resection rate and length of stay showed no significant differences between matched groups (all *p* ≥ 0.20; most SMD < 0.10). These findings ensure that postoperative body composition differences were not attributable to disparities in operative complexity or invasiveness.

### 3.3 Impact of TDR on Recovery Patterns of Body Composition Metrics

**FIGURE 2:**
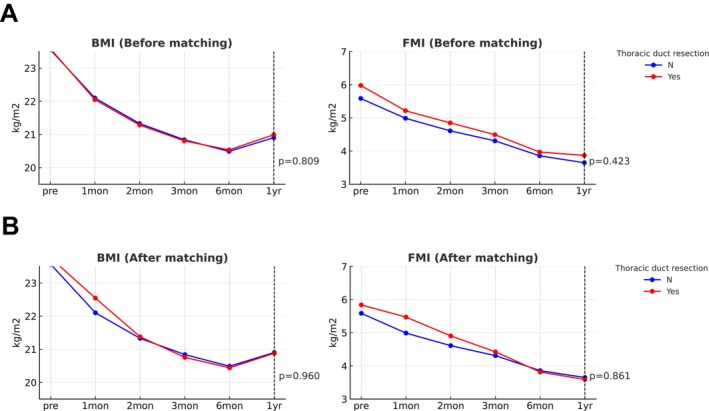
Temporal changes in adiposity‐related body composition metrics during the first postoperative year before and after propensity score matching. (A) Changes in body mass index (BMI) and fat mass index (FMI) over 12 months after oesophagectomy in the thoracic duct preservation and resection groups in the unmatched cohort. (B) Changes in BMI and FMI over 12 months in the 1:1 propensity score–matched cohort, showing similar temporal trajectories between the two groups.

A total of 1925 BIA measurements were obtained from 347 patients across six standardized time points, allowing detailed temporal analysis of both adiposity‐ and sarcopenia‐related indices. Non‐sarcopenia indices, including BMI and FMI, exhibited a gradual decline over the 12 months following oesophagectomy, with no significant differences observed between the TDR and preservation groups (*p* = 0.809 for BMI, *p* = 0.423 for FMI; Figure 2A). After PSM, BMI and FMI trajectories remained similar between the two groups throughout the postoperative year (Figure 2B).

**FIGURE 3:**
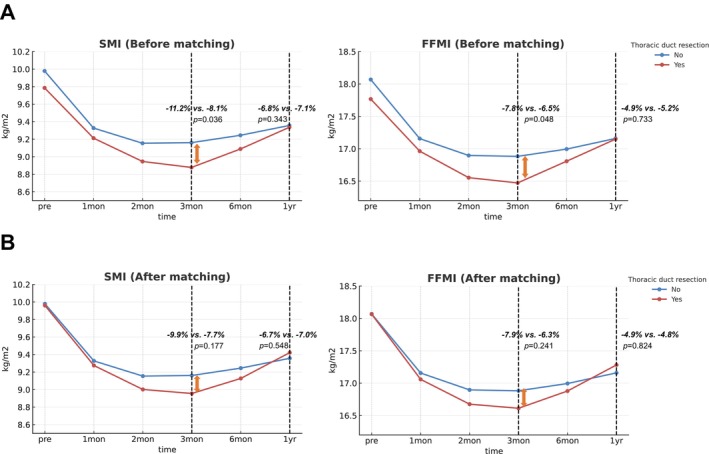
Temporal changes in muscle‐related body composition metrics during the first postoperative year before and after propensity score matching. (A) Changes in skeletal muscle index (SMI) and fat‐free mass index (FFMI) over 12 months after oesophagectomy in the thoracic duct preservation and resection groups in the unmatched cohort. (B) Changes in SMI and FFMI over 12 months in the 1:1 propensity score–matched cohort, showing an early postoperative decline followed by gradual recovery in both groups, with similar overall patterns over 12 months.

In contrast, sarcopenia‐related metrics—SMI and FFMI—declined sharply during the early postoperative period, followed by gradual recovery beginning around the third month. Although both groups reached comparable levels by 12 months (*p* = 0.343 for SMI, *p* = 0.733 for FFMI), patients in the TDR group experienced significantly greater reductions in SMI and FFMI within the first 3 months postoperatively (*p* = 0.036 for SMI, *p* = 0.048 for FFMI; Figure 3A). At 3 months, the TDR group showed a median reduction of 11.2% in SMI and 7.8% in FFMI from baseline. However, the recovery trajectories in this group paralleled those of the preservation group by 6 months, indicating that the adverse effects of TDR on muscle and fat‐free mass are likely transient. After PSM, the early postoperative decline in SMI and FFMI also persisted numerically in the TDR group; however, these differences were no longer statistically significant at 3 months (SMI, *p* = 0.177; FFMI, *p* = 0.241; Figure 3B). By 12 months, matched patients in both groups exhibited comparable SMI and FFMI recovery, reinforcing that the early disadvantages associated with TDR are transient and not sustained over the long term.

### 3.4 Subgroup Analysis in Clinical Stage I Patients

**FIGURE 4:**
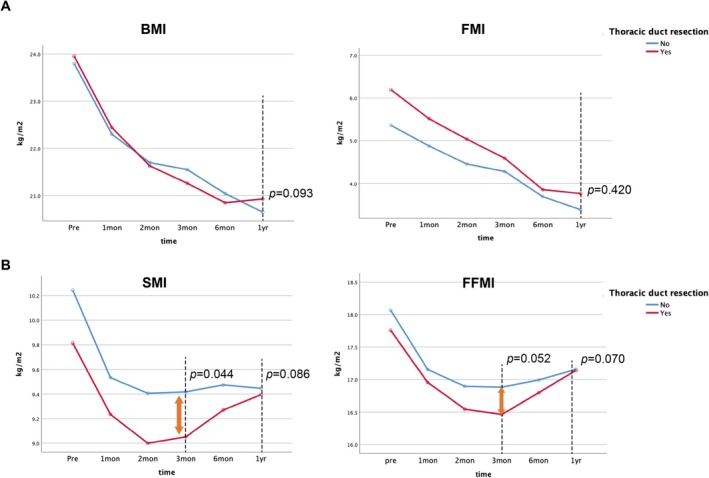
Subgroup analysis of body composition metrics in clinical stage I patients. (A) Changes in skeletal muscle mass index (SMI) and fat‐free mass index (FFMI) during the first 3 postoperative months in the TDR and preservation groups. (B) SMI and FFMI at each time point up to 12 months postoperatively in the TDR and preservation groups.

To further minimize selection bias and isolate the effect of TDR from the confounding influence of tumour burden and cancer‐related cachexia often observed in advanced stages, we performed a separate analysis limited to patients with clinical stage I disease. In this subgroup, trends in body composition metrics mirrored those of the overall cohort. Whereas early‐stage patients generally experienced milder postoperative declines, the TDR group still showed a significantly steeper reduction in SMI (median reduction, −8.4%) and FFMI during the first 3 months compared to the preservation group (Figure 4A). Despite this initial decline, muscle mass recovery began around postoperative months 2–3, and by 12 months, SMI and FFMI levels were comparable between groups (Figure 4B). These findings suggest that even in early‐stage ESCC, TDR may contribute to transient sarcopenia, although the effects are not sustained beyond the first postoperative year.

### 3.5 Exploratory Analysis of Nutritional Laboratory Markers

Baseline nutritional markers—including albumin, total lymphocyte count, total cholesterol, PNI and CONUT—were comparable between the TDR and preservation groups (all *p* ≥ 0.20; Figures S1 and S2). During the first postoperative year, both groups demonstrated a similar pattern of early postoperative decline followed by partial recovery in albumin and PNI. At 1–3 months, postoperative values were not significantly different between the TDR and preservation groups for albumin (4.15 vs. 4.14 g/dL, *p* = 0.399), total cholesterol (175 vs. 178 mg/dL, *p* = 0.462), lymphocyte count (1820 vs. 1790/mm^3^, *p* = 0.411), PNI (51.0 vs. 50.7, *p* = 0.374) or CONUT score (1.2 vs. 1.3, *p* = 0.588). Similarly, at 6–12 months, values for albumin (4.27 vs. 4.38 g/dL, *p* = 0.970), total cholesterol (181 vs. 186 mg/dL, *p* = 0.552), lymphocyte count (1960 vs. 2040/mm^3^, *p* = 0.365), PNI (52.1 vs. 52.8, *p* = 0.442) and CONUT score (1.1 vs. 1.2, *p* = 0.603) remained comparable between the two groups.

## 4 Discussion

Our study demonstrates that TDR, while traditionally performed to enhance locoregional control during radical oesophagectomy, does not result in sustained deterioration of body composition. Although TDR was associated with a sharper early decline in sarcopenia‐related indices—namely, SMI and FFMI—these effects were transient. Muscle mass recovery began around postoperative month 3, and normalization was observed by 12 months. These findings suggest that TDR, when oncologically justified, can be performed without lasting nutritional or functional compromise.

Although a few prior studies have explored the physiological effects of TDR, including those by Nishimura et al. [8] (*n* = 217), Fujisawa et al. [9] (*n* = 174) and Aiko et al. [7] (*n* = 39), most have been limited by small sample sizes, heterogeneous methodologies and the lack of detailed temporal assessment within the first postoperative year. In contrast, our study uniquely evaluated body composition changes at 1, 2, 3, 6 and 12 months, allowing for a more nuanced understanding of early and intermediate recovery trajectories. Moreover, with a cohort of over 300 patients, it represents the largest dataset to date on this topic. Body composition was assessed using BIA, a validated and reproducible tool that enables precise quantification of skeletal muscle and fat mass, thereby enhancing the accuracy and clinical relevance of our findings. In addition, the overall recovery patterns—including the early but reversible decline in muscle‐related indices—were directionally consistent in the matched cohort, supporting the robustness of the observed trajectories.

The sharper initial decline in muscle‐related metrics in the TDR group highlights the importance of proactive perioperative management [11]. Early, structured interventions—such as individualized nutritional supplementation, targeted physical rehabilitation and close postoperative monitoring—may mitigate transient sarcopenia and facilitate timely functional recovery [12]. These strategies are particularly vital during the first 3 months after surgery, when vulnerability to muscle loss is greatest.

Subgroup analysis of clinical stage I patients further supports the hypothesis that muscle loss following TDR is driven by the procedure itself, rather than cancer‐related cachexia. Despite their minimal oncologic stress and low risk of cancer‐associated cachexia [13], Stage I patients experienced similar early declines in SMI and FFMI. In this context—where the oncologic benefit of TDR may be marginal—the potential functional trade‐offs must be carefully weighed. For otherwise low‐risk patients, surgical decision‐making should be individualized, and perioperative support proactively implemented to preserve physical resilience and optimize long‐term outcomes [14].

The greater early decline in sarcopenia‐related indices after TDR may reflect transient lymphatic interruption rather than differences in surgical invasiveness. Although systemic nutritional markers—including albumin, lymphocyte count, lipid profiles, PNI and CONUT—were comparable between groups, thoracic duct removal can temporarily reduce chyle flow and the delivery of lipid and protein substrates essential for muscle maintenance. This short‐lived disruption in lymphatic nutrient transport aligns with the timing of postoperative muscle loss observed in our cohort, which peaked within the first 2–3 months. Importantly, this decline occurred despite uniform postoperative nutritional support, suggesting that the early deficit is more likely procedure related than driven by systemic malnutrition.

From a physiological perspective, the absence of long‐term nutritional impairment following TDR may be explained by compensatory lymphatic mechanisms [15]. After thoracic duct ligation or resection, alternative lymphatic pathways—including mediastinal, intercostal and abdominal collaterals—can undergo adaptive remodelling to maintain lymphatic return [16]. In some cases, direct absorption of chyle into systemic venous circulation, such as the azygos system or inferior vena cava, has also been described [15, 16, 17]. These adaptive mechanisms are consistent with the recovery of SMI and FFMI after postoperative month three and their near‐complete normalization by 1 year. Collectively, these observations suggest that TDR‐related early sarcopenia reflects transient, localized lymphatic disturbance rather than persistent systemic nutritional compromise.

Despite its strengths, this study has several limitations. Its retrospective design and reliance on BIA—which can be influenced by fluid status—may limit measurement precision. Future studies using more accurate body composition tools, such as dual‐energy x‐ray absorptiometry (DEXA) or CT‐based volumetrics, along with extended follow‐up, would enhance the validity of these findings [18, 19]. Additionally, although PSM improved baseline comparability, the reduced matched sample size may have limited the statistical power to detect early between‐group differences. Multicentre studies with larger cohorts are warranted to increase generalizability and to identify patient subgroups who may benefit most from targeted recovery interventions following TDR. Finally, because nutritional laboratory data were incomplete and not collected in a standardized manner, our ability to fully delineate the physiological mechanisms underlying the transient sarcopenia observed after TDR was limited, highlighting the need for more comprehensive, protocolized evaluations in future research.

In conclusion, TDR during radical oesophagectomy does not produce long‐term detriment to body composition or nutritional status, affirming its safety from a functional standpoint. However, the temporary impact on muscle mass—especially in early‐stage patients—warrants thoughtful perioperative planning. Tailored rehabilitation and nutritional interventions can help accelerate recovery, reduce sarcopenia‐related morbidity and ensure optimal postoperative outcomes. These findings underscore the importance of individualized care and dynamic monitoring strategies in the modern surgical management of oesophageal cancer.

## Funding

The authors have nothing to report.

## Conflicts of Interest

The authors declare no conflicts of interest.

## Supporting information


**Figure S1:** Longitudinal changes in serum albumin levels according to thoracic duct resection.
**Figure S2:** Longitudinal changes in nutritional laboratory and composite indices according to thoracic duct resection. (A) Total cholesterol level. (B) Total lymphocyte count. (C) Prognostic nutrition index (PNI). (D) Controlling Nutritional Status (CONUT) score.

## Data Availability

The data supporting the findings of this study are available from the corresponding author upon reasonable request.

## References

[1] P. C. Wu and M. C. Posner , “The Role of Surgery in the Management of Oesophageal Cancer,” Lancet Oncology 4, no. 8 (2003): 481–488, 10.1016/s1470-2045(03)01167-7. 12901962

[2] H. Akiyama , M. Tsurumaru , H. Udagawa , and Y. Kajiyama , “Radical Lymph Node Dissection for Cancer of the Thoracic Esophagus,” Annals of Surgery 220 (1994): 364–372, 10.1097/00000658-199409000-00012. 8092902 PMC1234394

[3] S. Matsuda , H. Kawakubo , H. Takeuchi , et al., “Minimally Invasive Oesophagectomy With Extended Lymph Node Dissection and Thoracic Duct Resection for Early‐Stage Oesophageal Squamous Cell Carcinoma,” British Journal of Surgery 107 (2020): 705–711, 10.1002/bjs.11487. 32077101

[4] S. Matsuda , H. Takeuchi , H. Kawakubo , N. Ando , and Y. Kitagawa , “Current Advancement in Multidisciplinary Treatment for Resectable cStage II/III Esophageal Squamous Cell Carcinoma in Japan,” Annals of Thoracic and Cardiovascular Surgery 22 (2016): 275–283, 10.5761/atcs.ra.16-00111. 27384595 PMC5088392

[5] S. Matsuda , M. Takeuchi , H. Kawakubo , H. Takeuchi , and Y. Kitagawa , “Oncological and Physiological Impact of Thoracic Duct Resection in Esophageal Cancer,” Diseases of the Esophagus 36 (2023): doad015, 10.1093/dote/doad015. 36950928 PMC10543365

[6] M. Imamura , Y. Shimada , T. Kanda , et al., “Hemodynamic Changes After Resection of Thoracic Duct for En Bloc Resection of Esophageal Cancer,” Surgery Today 22 (1992): 226–232, 10.1007/bf00308827. 1392326

[7] S. Aiko , Y. Yoshizumi , T. Matsuyama , Y. Sugiura , and T. Maehara , “Influences of Thoracic Duct Blockage on Early Enteral Nutrition for Patients Who Underwent Esophageal Cancer Surgery,” Japanese Journal of Thoracic and Cardiovascular Surgery 51 (2003): 263–271, 10.1007/bf02719376. 12892455

[8] E. Nishimura , S. Matsuda , H. Kawakubo , et al., “The Impact of Thoracic Duct Resection on the Long‐Term Body Composition of Patients Who Underwent Esophagectomy for Esophageal Cancer and Survived Without Recurrence,” Diseases of the Esophagus 36 (2023): doad002, 10.1093/dote/doad002. 37465862 PMC10473448

[9] K. Fujisawa , Y. Ohkura , M. Ueno , A. Yago , H. Shimoyama , and H. Udagawa , “Nutritional Outcomes of Thoracic Duct Resection for Radical Esophagectomy by Assessing Body Composition Changes in One Year: A Single‐Center Retrospective Study,” Annals of Surgical Oncology 28 (2021): 8414–8425, 10.1245/s10434-021-10222-8. 34085142

[10] H. E. Kim , Y. H. Yang , B. J. Park , S. Y. Park , I. K. Min , and D. J. Kim , “Skeletonizing En Bloc Esophagectomy Revisited: Oncologic Outcome in Association With the Presence of Thoracic Duct Lymph Nodes,” Annals of Surgical Oncology 29 (2022): 4909–4917, 10.1245/s10434-022-11496-2. 35438467

[11] A. Weimann , M. Braga , F. Carli , et al., “ESPEN Guideline: Clinical Nutrition in Surgery,” Clinical Nutrition 36 (2017): 623–650, 10.1016/j.clnu.2017.02.013. 28385477

[12] F. Carli and C. Scheede‐Bergdahl , “Prehabilitation to Enhance Perioperative Care,” Anesthesiology Clinics 33 (2015): 17–33, 10.1016/j.anclin.2014.11.002. 25701926

[13] P. van Hagen , M. C. Hulshof , J. J. van Lanschot , et al., “Preoperative Chemoradiotherapy for Esophageal or Junctional Cancer,” New England Journal of Medicine 366 (2012): 2074–2084, 10.1056/NEJMoa1112088. 22646630

[14] C. Gillis and F. Carli , “Promoting Perioperative Metabolic and Nutritional Care,” Anesthesiology 123 (2015): 1455–1472, 10.1097/aln.0000000000000795. 26248016

[15] G. Hidden , P. Menard , and J. Y. Zorn , “Lymphaticovenous Communications. Role of the Lymph Nodes,” Anatomia Clinica 7 (1985): 83–91, 10.1007/bf01655509. 4041277

[16] K. Phang , M. Bowman , A. Phillips , and J. Windsor , “Review of Thoracic Duct Anatomical Variations and Clinical Implications,” Clinical Anatomy 27 (2014): 637–644, 10.1002/ca.22337. 24302465

[17] R. Bhardwaj , H. Vaziri , A. Gautam , E. Ballesteros , D. Karimeddini , and G. Y. Wu , “Chylous Ascites: A Review of Pathogenesis, Diagnosis and Treatment,” Journal of Clinical and Translational Hepatology 6 (2018): 105–113, 10.14218/jcth.2017.00035. 29577037 PMC5863006

[18] C. M. Prado and S. B. Heymsfield , “Lean Tissue Imaging: A New Era for Nutritional Assessment and Intervention,” JPEN Journal of Parenteral and Enteral Nutrition 38 (2014): 940–953, 10.1177/0148607114550189. 25239112 PMC4361695

[19] M. Mourtzakis , C. M. Prado , J. R. Lieffers , T. Reiman , L. J. McCargar , and V. E. Baracos , “A Practical and Precise Approach to Quantification of Body Composition in Cancer Patients Using Computed Tomography Images Acquired During Routine Care,” Applied Physiology, Nutrition, and Metabolism 33 (2008): 997–1006, 10.1139/h08-075. 18923576

