# Supplementary material for: Impact of Thoracic Duct Resection on Postoperative Body Composition Trajectory After Oesophagectomy: A Prospective Cohort Study
Source: J Cachexia Sarcopenia Muscle. 2026 Feb 10;17(1):e70209. doi: 10.1002/jcsm.70209 (PMC12891974; doi:10.1002/jcsm.70209)

**Supplemental Online Content**

Hong TH, Yang YH, Kim HE et al. *Impact of Thoracic Duct Resection on Postoperative Body Composition Trajectory After Esophagectomy: A Prospective Cohort Study.*

**Supplementary Figure 1. Longitudinal Changes in Serum Albumin Levels According to Thoracic Duct Resection**

**
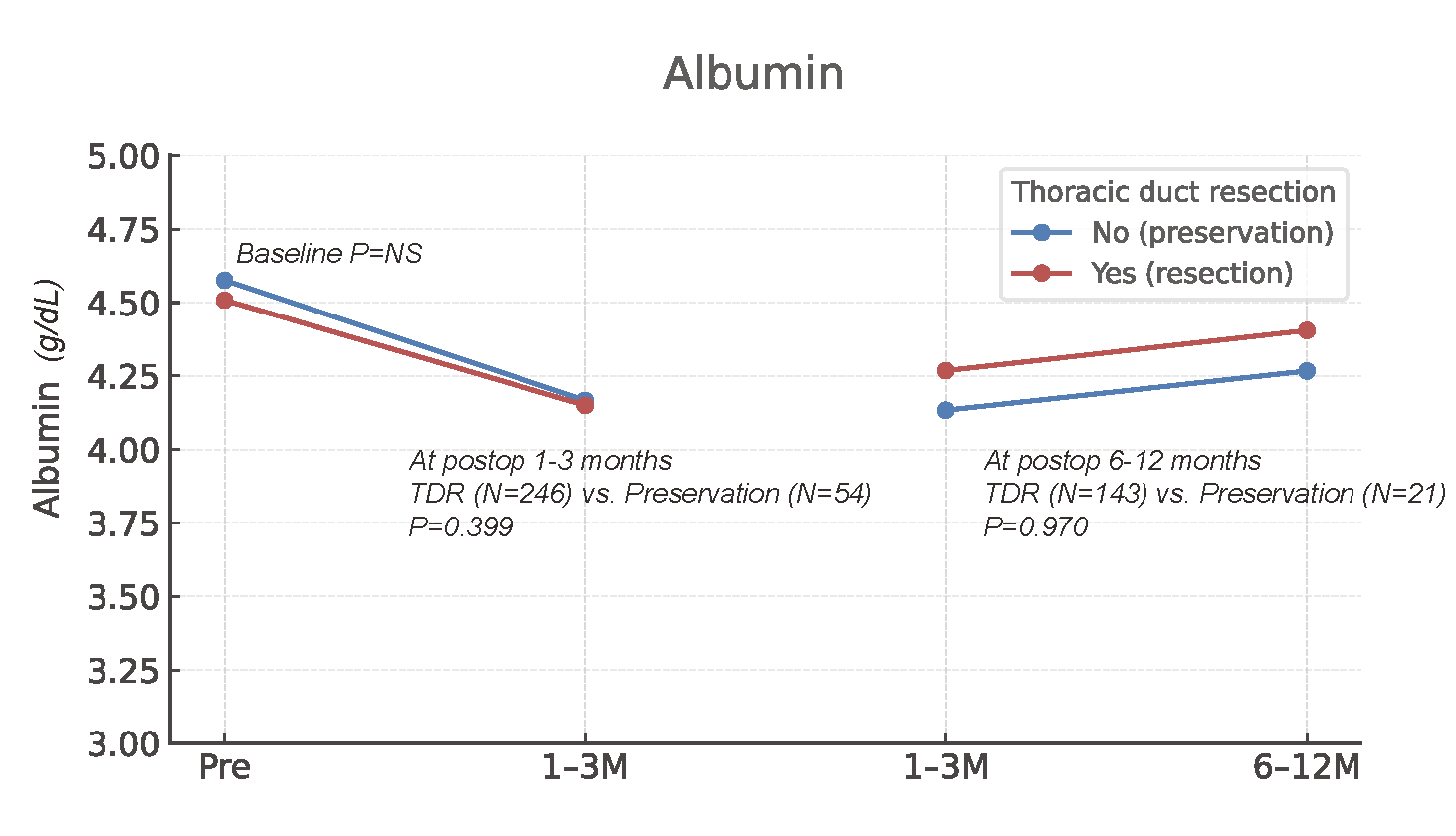
**

**Supplementary Figure 2. Longitudinal changes in nutritional laboratory and composite indices according to thoracic duct resection.** (A) Total Cholesterol Level. (B) Total Lymphocyte Count. (C) Prognostic Nutrition Index (PNI). (D) Controlling Nutritional Status (CONUT) score.


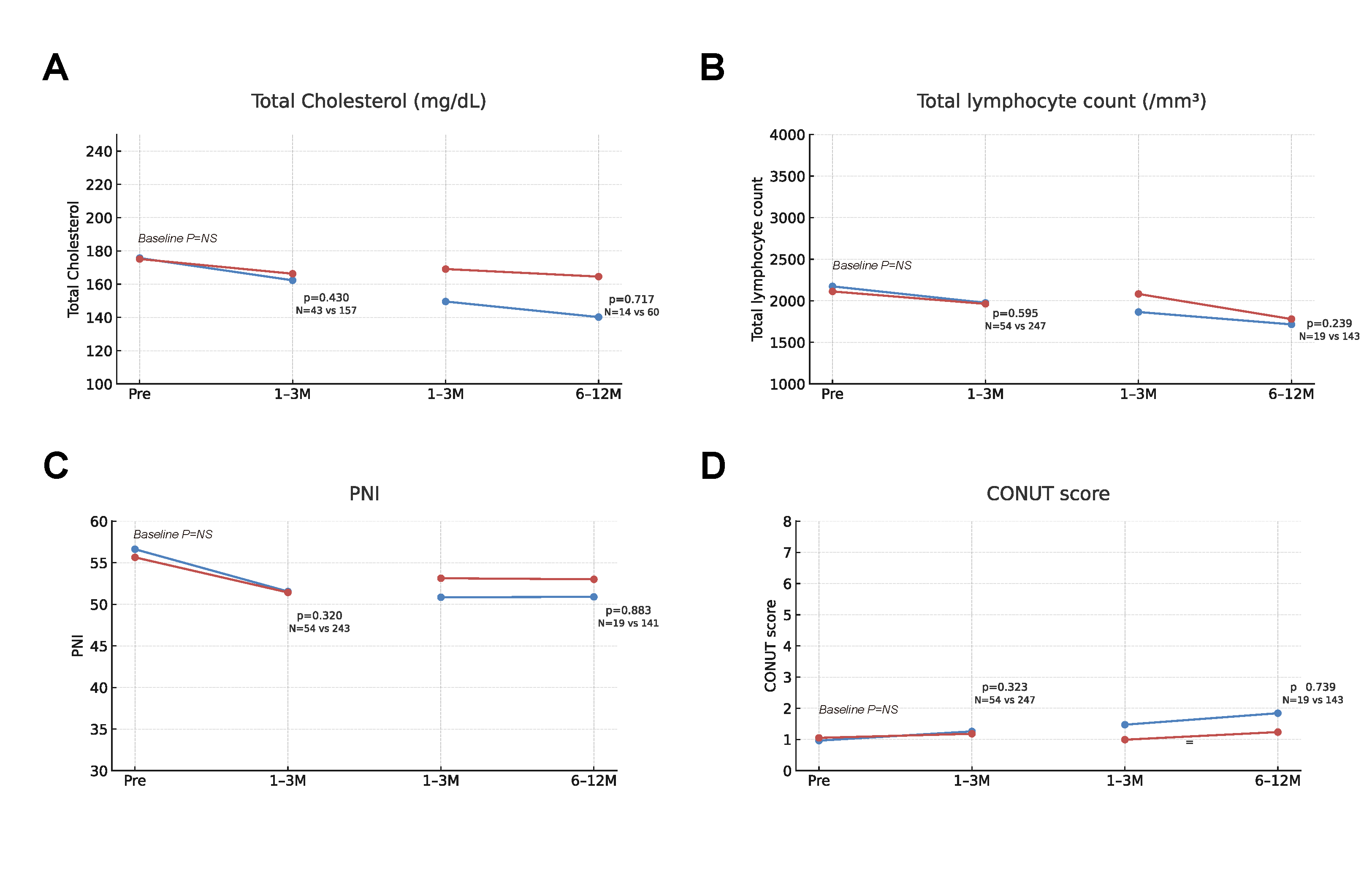

Supplement: Supplementary file 1 — Figure S1: Longitudinal changes in serum albumin levels according to thoracic duct resection. Figure S2: Longitudinal changes in nutritional laboratory and composite indices according to thoracic duct resection. (A) Total cholesterol level. (B) Total lymphocyte count. (C) Prognostic nutrition index (PNI). (D) Controlling Nutritional Status (CONUT) score. [file JCSM-17-e70209-s001.docx]
